# Measuring fragmentation in dissociative identity disorder: the integration measure and relationship to switching and time in therapy

**DOI:** 10.3402/ejpt.v5.22250

**Published:** 2014-01-03

**Authors:** M. Rose Barlow, James A. Chu

**Affiliations:** 1 Psychology Department, Boise State University, Boise, ID, USA; 2 McLean Hospital, Belmont, MA, USA; 3 Harvard Medical School, Department of Psychiatry, Boston, MA, USA

**Keywords:** Dissociative identity disorder, dissociation, alternate identity, integration measure, psychotherapy, awareness, measurement, betrayal trauma

## Abstract

**Background:**

Some people with dissociative identity disorder (DID) have very little communication or awareness among the parts of their identity, while others experience a great deal of cooperation among alternate identities. Previous research on this topic has been sparse. Currently, there is no empirical measure of integration versus fragmentation in a person with DID. In this study, we report the development of such a measure.

**Objective:**

The goal of this study was to pilot the integration measure (IM) and to address its psychometric properties and relationships to other measures. The IM is the first standardized measure of integration in DID.

**Method:**

Eleven women with DID participated in an experiment that included a variety of tasks. They filled out questionnaires about trauma and dissociation as well as the IM. They also provided verbal results about switching among alternate identities during the study sessions.

**Results:**

Participants switched among identities an average of 5.8 times during the first session, and switching was highly correlated with trauma. Integration was related to switching, though this relationship may be non-linear. Integration was not related to time in psychotherapy.

**Conclusions:**

The IM provides a useful beginning to quantify and study integration and fragmentation in DID. Directions for future research are also discussed, including expanding the IM from this pilot. The IM may be useful in treatment settings to assess progress or change over time.

Dissociative identity disorder (DID) is defined in DSM-5 as the presence of two or more distinct personality states, and it specifies that dissociative symptoms such as discontinuity in self or consciousness (i.e., switching) may either be observed by others or reported by the individual herself (American Psychiatric Association, 2013). The *Guidelines for Treating Dissociative Identity Disorder in Adults* of the International Society for the Study of Dissociation (2011) use the term “alternate identity,” which we will use in this paper for continuity.

Cognitive and neurological evidence demonstrates that alternate identities in DID have some qualities of separate individuals, but they are not completely separate from each other. Putnam (1997) argued that alternate identities are discrete states of consciousness that are demonstrably dissociated from each other (different respiration rates, muscle tone, etc.). Reinders and colleagues, among other researchers, have demonstrated different patterns of neural network activation and cerebral blood flow between alternate identities (Reinders et al., 2006; Reinders, Willemsen, Vos, den Boer, & Nijenhuis, 2012). They also propose that fragmentation of information storage may occur only when that information is personally relevant, or when attempting to transfer information between different types of identities that serve different roles.

Previous research examining why and how persons with DID switch among their alternate identities is sparse. Putnam (1994) reported that certain alternate identities are more likely to come out when preceded by specific others, and that some identities have a tendency to be followed by specific others. Moreover, Putnam (1994) also reported preliminary results showing that each alternate identity's physiology may be affected by the identities that precede him or her, making it difficult to determine what should be regarded as a baseline measurement and therefore potentially confounding results. Putnam (1988) appears not to have followed up on this intriguing finding or methodological application, having published only one empirical paper about switching. Switching between alternate identities is one strategy DID patients may use to block out ongoing awareness of unwanted information (Elzinga, Phaf, Ardon, & van Dyck, 2003), and dissociative responses in general may help regulate responses to potentially frightening information (Dalenberg et al., 2012). More research is needed to understand the dynamics and mechanics of fragmentation and integration. One unique aspect of the current research is the preliminary development of the first standard instrument to measure integration.

Complete integration is the fusion of all identities into one personality and is a somewhat controversial topic. It is often a lengthy and subtle process, but a few studies have begun to show that effective treatment leads to stable integration (e.g., Coons & Bowman, 2001). Integration is associated with a wide variety of benefits, including reduction of dissociative symptoms and other associated symptoms related to DID (Coons & Bowman, 2001; Kluft, 1993).

The longest follow-up study of DID patients (Coons & Bowman, 2001) has shown that integration can be sustained for over 10 years, suggesting that the benefits of effective treatment may last indefinitely. Simpler cases (i.e., fewer identities) have briefer treatments, but even in complex cases, the majority of patients see improvements in their functioning during the course of therapy (Brand et al., 2013; Maldonado, Butler, & Spiegel, 1998; Putnam, 1995). These benefits include lowered symptoms of many types, decreased suicidality, decreased illicit drug use, and greatly decreased hospitalization, even without complete integration (Brand, Classen, Lanius et al., 2009; Brand, Classen, McNary, & Zaveri, 2009). Treatment for dissociative disorders also leads to improved functioning and more endorsement of “feeling good” in an ongoing longitudinal study, particularly when clinicians are well trained (Brand et al., 2013). There is still a need for more treatment outcome studies; however, an increasing number of studies are showing that DID can be successfully treated, once the correct diagnosis is made and the client's condition is understood. The treatment process is frequently long and complex, even for experts (Brand, Classen, Lanius et al., 2009). Treatment did not decrease dissociation levels significantly over a 30-month follow-up, though dissociation did decline slightly; some patients improved while others did not, and most continued to have daily psychiatric problems (Brand et al., 2013). Given the complexity of treatment courses with these polysymptomatic patients, more tools are needed to look at changes in functioning across alternate identities.

## Rationale for current study

Currently, there is no existing empirical measure of integration versus fragmentation in a person with DID. Because DID is believed to be developed in order to hold various parts of memory away from each other (e.g., Dalenberg et al., 2012), experimenters who assess memory functioning must necessarily be concerned with the pattern and extent of fragmentation and integration in their participants. There are clinician and patient reports describing degrees and components of integration qualitatively, but few use the same vocabulary and almost none use the same scale. None have attempted to quantify integration.

Unlike in previous research, in this study the participants were not asked to switch at certain times nor to certain identities but rather were given their choice over when and whether to switch. Many studies of DID have required that participants be able and willing to switch to certain pre-specified identities at will and to regulate how long each of those identities remains in control of the body. Such participants may not be representative of most people with DID. Therefore, we recruited without regard for this skill.

The goal of this study was to pilot the integration measure (IM) and to address its psychometric properties and relationships to other measures. The current research represents a step forward for the field of dissociation studies by its development of a new scale. This measure will be useful for both researchers and clinicians. For example, researchers could use it to assess whether asking participants to switch parts of their personalities on request is feasible. Clinicians could use this instrument to assess change over the course of treatment, particularly in a progression toward eventual integration of the personality parts. We predicted that higher levels of trauma would be linked with higher levels of dissociation and switching, and with lower IM scores. We predicted that years in therapy would be negatively correlated with the IM score. We also hypothesized that switching would be related to scores on the IM, possibly in a non-linear way. Based on previous research, very fragmented people should report many switches exactly because they are so fragmented. With increasing integration should come an increasing awareness of the switches, which would show up as more self-report of switching.

## Method

### Participants

*Recruiting*. The first type of recruitment took place at a psychiatric hospital in the Boston area and resulted in one inpatient participant. No contacted patients refused to participate; low recruitment was due to potential participants being released from the hospital before they could be contacted. The second type of (outpatient) recruitment took place in the Eugene and Portland areas of Oregon and the Seattle area of Washington. A letter explaining the project was sent to clinicians and treatment centers that specialized in dissociation or trauma. The letter asked clinicians to have interested clients with a diagnosis of DID contact the experimenter. Information was also sent out over an electronic mailing list, and a website provided details about the study. No potential participants refused to participate once enrolled in the study. We do not have data about what percentage of people who had access to the information chose to contact us.

*Demographics*. The participants consisted of 11 women who had been diagnosed with DID. One participant did not answer demographic questions. The mean age was 35.35 years (*SD*=12.57, range=23–62). Seven participants reported being currently on psychiatric medications, mostly antidepressants. The participant group had one member with a high school education or General Educational Development, four members with some college or technical school education, two members who had completed college or technical school, two members with some graduate school education, and one member who had completed graduate school. Participants had been hospitalized an average of 3.6 times (range=0–15 times) and had been in therapy for an average of 12.05 years (*SD*=6.73, range=2–20 years). One participant had been diagnosed with DID between 1 and 3 years ago, four had been diagnosed 3–6 years ago, and four had been diagnosed more than 6 years ago (two participants did not answer).

## Materials

*Demographics*. The demographics form asked basic open-ended questions such as age and gender, level of education, job, psychiatric medication, and amount of psychotherapy and prior hospitalization.

*Dissociative Experiences Scale (DES;* Bernstein & Putnam, 1986 *)*. The DES is a 28-item self-report measure that is used to assess different types of dissociative experiences. It has been used extensively with a wide range of populations in countries around the world and has been found to have strong reliability and validity (see Briere, 1997; Carlson & Putnam, 1993 for reviews). Although the DES was not designed for diagnostic use, it can distinguish populations of DID from those with other psychiatric and dissociative disorders (Bernstein & Putnam, 1986; Van IJzendoorn & Schuengel, 1996). Carlson and Putnam (1993) reported that the DES has a sensitivity rate of 74% and a specificity rate of 80%, and also report that other studies find similar numbers. Adults in the general population usually score below 10 (Bernstein & Putnam, 1986; Carlson & Putnam, 1993). Van IJzendoorn and Schuengel (1996))reported that the mean alpha reliability of the DES in their meta-analysis was 0.93. Test–retest reliability has been reported to range from 0.79 to 0.96 and split-half reliability as 0.83 (Carlson & Putnam, 1993).

*Brief Betrayal Trauma Survey (BBTS;* Goldberg & Freyd, 2006 *)*. This measure is designed to measure the frequency of various traumatic events, including natural disasters, sexual trauma, physical and emotional abuse, and witnessing violence. It distinguishes between interpersonal trauma inflicted by someone with whom the victim was close to (betrayal trauma) and the same trauma committed by not-close perpetrators. Each item asks the participant to circle the number of times an event has happened before age 18 and after age 18. Possible responses ranged from *never*, coded as 0, to *more than 100 times*, coded as 5. The BBTS has good test–retest reliability (Goldberg & Freyd, 2006). An example of a low-betrayal item is “You were in a major earthquake, fire, flood, hurricane, or tornado that resulted in significant loss of personal property, serious injury to yourself or a significant other, the death of a significant other, or the fear of your own death.” An item that is high in interpersonal betrayal (betrayal trauma) is “You were made to have some form of sexual contact, such as touching or penetration, by someone with whom you were very close (such as a parent or lover).” In our sample, the split-half reliability was 0.98 and the Cronbach's alpha was 0.93.

*Integration measure (IM)*. This questionnaire is a first attempt to measure components of integration, such as awareness of other alternate identities, communication between identities, shared executive control, and co-consciousness. Two of these elements, internal communication and co-consciousness, are mentioned as precursors of integration in an early article based on clinical experience (Greaves, 1989). The IM was developed as a collaboration between the authors of this paper. The questions were based on both collaborators’ understanding of current research, as well as clinical experience. Higher scores imply more co-consciousness, shared awareness, communication, and cooperation among the identities, and thus less fragmentation.

Possible integration scores range from 0 to 20, with 0 indicating almost total fragmentation with little or no communication or awareness among identities, and 20 indicating almost total integration, with all parts aware of and cooperating with each other. The IM includes both multiple-choice and open-ended questions. Items 5 through 9 provided the clearest answers and the most coherent measure of integration itself, as opposed to measuring co-consciousness or executive control. These five items, called “integration,” were therefore the only ones analyzed for the current study. In the Supplementary file, the numbers next to each possible response indicate the scoring for these items.

## Procedure

*Session 1*. Participants who were from the Eugene, Oregon area, came into a university psychology laboratory for both sessions. Other participants in the Pacific Northwest region participated, by their choice, in their own homes or in the home of a friend and fellow-participant (in which case, participants did not talk with one another between their sessions). One woman participated in an office in the building where she was attending residential psychiatric treatment. All participants had individual experimental sessions. Participation was coded with an anonymous code number. All participants gave informed consent.

Participants completed multiple cognitive tasks relating to another study (Barlow, 2011). Tasks were videotaped for later coding. All participants were given both an oral and a written debriefing, and were paid. The first session usually took less than 70 min.

During this session, participants did not receive any instructions regarding switching among their alternate identities. All but one of the participants later reported that they had switched several times during the experiment; some participants switched quite visibly, though not all did. For example, some participants would stop speaking, blink, look around, and then speak in a different tone of voice. Participants were paid $10 for each session.

*Session 2*. The details of meeting and informed consent were similar in the first and second sessions. Participants were seen individually. The primary purpose of the second session was to collect participants’ reports of what they had experienced in the first session. In order to aid participants’ recollection of the experiences, the second sessions took place in the same location as the first sessions, anytime from the next morning to a few days later. Participants had the option to view the videotape of themselves from the first session; however, all but three participants declined it.

Verbally and/or with the aid of the videotape, the experimenter reminded participants of each of the tasks they had performed in Session 1. For each task, participants verbally reported which identity performed each task, as well as whether there were other identities watching or listening internally, and whether they switched during the task. At the conclusion of the verbal interview, participants filled out several additional questionnaires. As in Session 1, all participants were given both an oral and a written debriefing, and were paid. The second session took 40–90 min.

## Results

### Trauma and dissociation

All tests reported in this paper are two-tailed except where noted. Participants had a mean DES score of 56.16 (*SD*=21.88), which is consistent with DID populations and well above the normal range. Participants also reported very high levels of trauma on the BBTS, particularly trauma before age 18. Mean score on the BBTS for trauma before age 18 was 31.40 (*SD*=13.05). For high-betrayal trauma before age 18, the mean score was 13.00 (*SD*=3.37). Participants had a mean score of 19.10 (*SD*=10.84) for all trauma after age 18. For high-betrayal trauma after age 18, the mean score was 9.20 (*SD*=4.57). These levels of reported trauma are much higher than those reported by young adult populations (see Barlow, 2011 for comparison results).

### Switching

Participants self-reported switching anywhere from 0 to 12 times during the first session, coded conservatively and not counting co-consciousness. The mean number of switches reported in Session 1 was 5.8 (*SD*=3.60). The experimenter also observed switching during Session 2, but this session was not coded for switching. Dissociation was moderately correlated with switching (*r*=0.32, *p*=0.168, one-tailed, *n.s*.). Childhood high-betrayal trauma was positively correlated with switching (*r*=0.52, *p*=0.121, *n.s*.), and lifetime betrayal trauma was positively and strongly correlated (*r*=0.76, *p*<0.05). Switching was not correlated with the number of years in therapy. Post-hoc regression analyses revealed that no other variables in the broader study predicted switching as well as did lifetime betrayal trauma.

### Integration measure

This group of DID participants had a mean score of 6.54 on these items, with a standard deviation of 3.39. Based on their answers to the IM, two of the participants were noticeably more fragmented than the others. One of these two participants reported having been in therapy for only 3 years, while the other reported having been in therapy for 15 years, which was closer to the average for this group. The participant who had spent 3 years in therapy was also one of the participants most recently diagnosed with DID (1–3 years ago) but had never been hospitalized. Of the participants with the two highest (most integrated) scores, one was the inpatient and the other had been in therapy for 11 years, diagnosed more than 6 years ago.

On open-ended questions, participants reported having anywhere from 5 to 1,000 identities (mean=167.1, median=33, mode=5), with many answers being accompanied by much uncertainty (question marks, qualifying comments). Every participant reported that she knew there were identities she did not know about or was not aware of. Reports of how the identities communicated included sharing thoughts, hearing voices, sharing emotions and/or body sensations. Some reported communication through writing or drawing, or in dreams. On the final question, “Overall, how often do you feel you communicate and work together with your identities/parts?”, participant answers ranged from 4% of the time to 91% of the time (mean=54%, median=61%).

**Fig. 1:**
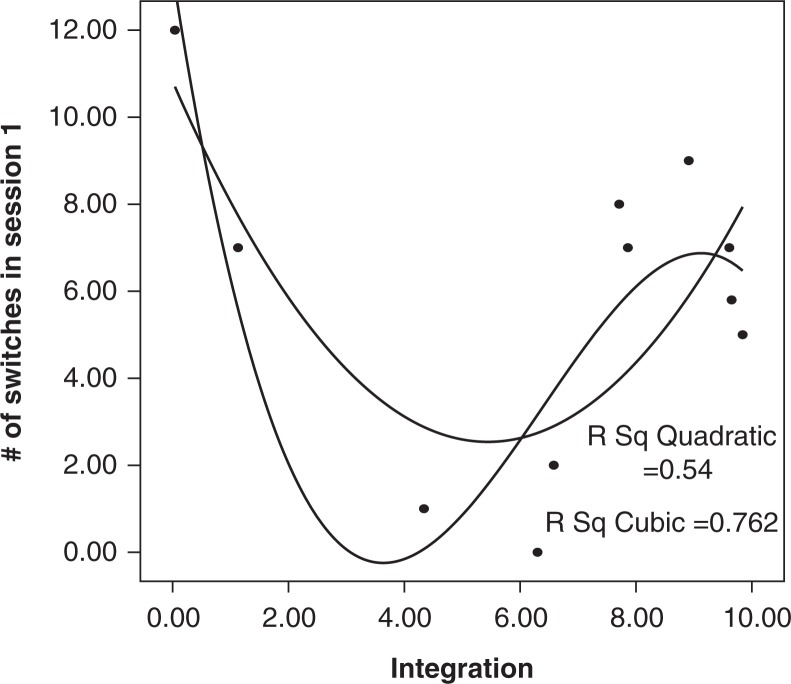
Relationship between integration score on integration measure and number of Session 1 switches.

Scores on the integration scale were related to number of reported switches in Session 1. Figure 1 is the scatterplot of this relationship with two fit lines overlaid, a quadratic and a cubic. A linear regression did not fit the data well (*R* ^2^=0.03; *F*(1, 9)=0.31, *p*=0.59, *n.s*.). The quadratic regression fits better and is significant with an *R* ^2^ of 0.54 (*F*(2, 8)=4.70, *p*=0.05). The cubic regression improves the fit with an *R* ^2^ of 0.76 (*F*(3, 7)=7.48, *p*<0.05). The *t*-test of integration^3^ was significant, indicating that adding this term did improve the fit over the quadratic regression (*t*= − 2.56, *p*<0.05). However, with the Bonferroni correction for multiple comparisons, the cubic regression becomes not statistically significant.

**Fig. 2:**
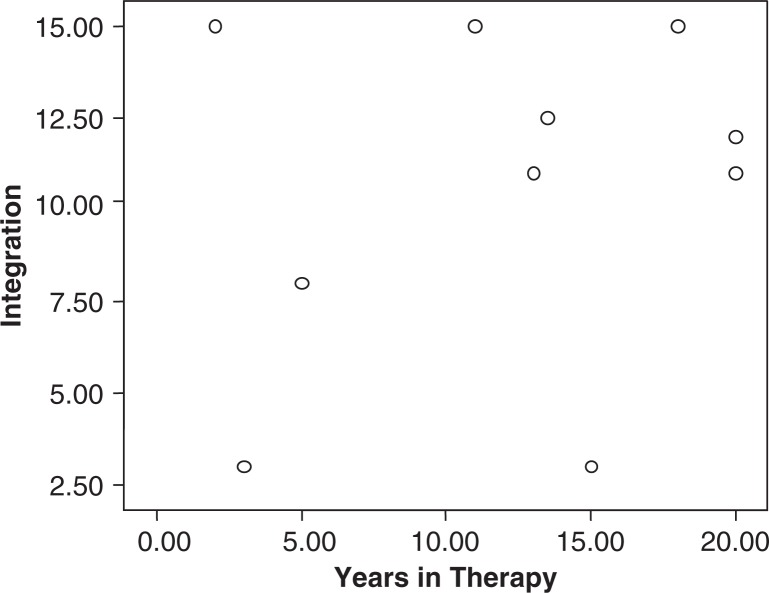
Relationship between years in therapy and integration.

Table 1 gives correlations among trauma variables, integration, and switching. The trauma variables were highly correlated with each other but were only slightly correlated with the integration score (*r*=0.22, *n.s*.). Figure 2 demonstrates that the relationship of therapy to integration is far from a simple one.

**Table 1:** Pearson's correlations among trauma, integration, and switching

	High-BT before age 18	High-BT after age 18	High-BT lifetime	Integration	No. of reported switches in Session 1
Child trauma	0.855**	0.226	0.567	−0.010	0.281
High-BT before age 18		0.499	0.819**	−0.241	0.523
High-BT after age 18			0.906**	−0.124	0.758*
High-BT lifetime				−0.200	
					−0.182

Note: High-BT stands for high-betrayal traumas such as sexual, physical, or emotional abuse perpetrated by someone to whom the victim was close.

** Correlation is statistically significant at the 0.01 level.

* Correlation is statistically significant at the 0.05 level.

## Discussion

Consistent with the etiological literature on DID, participants reported very high levels of all kinds of trauma that were measured, including trauma in childhood. Freyd's (1996) betrayal trauma theory proposes that for children who are abused and betrayed by the caregivers on whom they depend for attachment relationships, it is adaptive to remain unaware of the abuse in order to preserve the relationship. Dissociation is one way to keep relationship-threatening information out of conscious awareness and memory.

Switching between alternate identities has been reported to take anywhere from a few seconds (Putnam, 1988) to 30 sec (Tsai, Condie, Wu, & Chang, 1999) to brief times less than 2–5 min (Huntjens, Postma, Peters, Woertman, & van der Hart, 2003; Peters, Uyterlinde, Consemulder, & van der Hart, 1998; Putnam, 1988) to 10 min (Putnam, 1994). However, the switches that occurred during this study were rapid and appeared instantaneous. Some switches were readily apparent, while others were not, but for the most part the DID participants were able to identify when they had switched in the previous session. Future research is needed to examine this process more thoroughly.

The items on the IM were generated from a thorough knowledge of the DID literature and from extensive clinical experience. This pilot study marks the first time that this or any measure has attempted to quantify or classify integration in a standardized fashion. Although only items 5 through 9 were analyzed for this study, there is still much information to be gleaned from an examination of participants’ answers to the open-ended questions. Putnam (1994) suggested that during the switch process, participants’ abilities to observe stimuli, to learn, and to form new memories are impaired; more research is needed in this area.

Figure 2 demonstrates that the relationship of therapy to integration may be complex. Trauma was negatively associated with integration (the higher the trauma, the lower the integration), though this correlation was small. It is possible that there is a non-linear relationship between these variables, one that changes over time and from person to person. The present study did not ask about type or quality of therapy, only length. Very traumatized persons with complex DID may show less integration in spite of the length of treatment because they began with a greater degree of dissociation. In fact, even with therapy performed by clinicians who are experts in this field, most clients may not experience full integration at all, at least not in the time course studied so far (Brand et al., 2012). If the IM proves to have adequate test–retest reliability in the future, issues such as these can be addressed by using repeated testing throughout the course of therapy.

Scores on the integration subscale of the IM were related to the number of reported switches in Session 1 (see Fig. 1). Although this plot is based on only 11 participants, either a quadratic and a cubic relationship between these variables is plausible based on the previous literature about DID. The quadratic relationship would imply that there is a U-shaped curve such that very fragmented people report many switches exactly because they are so fragmented. Very integrated people with DID may report many switches because their increasing integration leads to an increasing awareness of the switches. People in the middle of the curve may have fewer switches than do these two groups because they are more controlled in their daily functioning than are the less integrated people, but do not have as much awareness of their switching as have the more integrated people. If this relationship between switching and integration is supported, it would also explain why the cubic curve fits so well. In a cubic relationship, the two variables would start with the quadratic relationship just described. The number of switches would then decrease toward zero as participants became more and more integrated, until eventually there were fewer identities to switch among, possibly leading to a single, fully integrated personality. However, interpretations such as these will have to wait for more data and replication before being supported.

### Limitations and future directions

This pilot study bases its conclusions on data collected from 11 participants with DID. Much of the previous research on DID has used samples of one to seven participants (e.g., Kong, Allen, & Glisky, 2008; Loewenstein, Hamilton, Alagna, Reid, & deVries, 1987; Nissen, Ross, Willingham, MacKenzie, & Schacter, 1988; Peters, et al., 1998; Schacter, Kihlstrom, Kihlstrom, & Berren, 1989; Tsai, et al., 1999), so having a sample this size is not unusual in research in this field. Reinders and colleagues (2006) also had 11 participants. However, a sample size of 11 does limit the number of inferential statistical tests that can meaningfully be performed. The small number of participants meant that observed power was very low in many of the analyses. In the future, dividing the concepts of interest into several smaller, more focused studies should alleviate some of these problems.

Another major limitation to this type of research is that, in measuring switching or integration among identities, we are much of the time measuring only *awareness* of these phenomena. Participants who dissociate their knowledge that they are dissociative cannot give accurate answers to survey questions on these topics. This limitation also affects other research on dissociation that relies on self-report data, particularly retrospective or overall self-report. Although assessing experiences retrospectively in the second session rather than at the time does present limitations, the first session was already long and somewhat stressful, and participant fatigue would have become a major factor had more tasks been added. In fact, Kong and colleagues (2008) found that retrospective self-report of dissociative experiences may show unanticipated patterns. Of their DID participants, the lowest DES scores were found in those who had been diagnosed in the distant past; they speculate that these scores result from “greater adjustment and acceptance of symptoms as a part of daily life” (p. 691). Despite this limitation, instruments such as the DES have long shown rich and useful results. While recognizing the limitations of our current measure, we hope that future researchers will find it useful when combined with other converging evidence regarding dissociative behavior.

The current pilot study is important in its development of the IM. Planned research in the future will revise and refine this measure, and will acquire norms that will enable the IM to distinguish among polyfragmented patients with DID, those with only a few identities, those who are fully integrated, and persons with other clinical conditions involving some degree of dissociation, such as dissociative disorder not otherwise specified, dissociative fugue, and borderline personality disorder. In addition, it is important to examine various types of alternate identities and the internal logic of fragmentation among the different types (such as emotion-based and logic-based). It would be useful to look at any relationship between the IM and the Progress in Treatment Questionnaire developed and used for dissociative clients in the work of Brand, Classen, Lanius, and colleagues (2009).

These initial pilot results are important additions to the sparse literature involving the scientific examination and measurement of switching and integration, especially as integration relates to years in therapy. Future research should address therapy variables, such as type of therapy, number of current diagnoses, therapeutic alliance, amount of time in therapy before receiving DID diagnosis, and so on, as well as examining the stability or change of IM scores over time. This area is rich with opportunities for future research that will illuminate the relationship between these complex variables.
